# Machine learning techniques to identify putative genes involved in nitrogen catabolite repression in the yeast *Saccharomyces cerevisiae*

**DOI:** 10.1186/1753-6561-2-s4-s5

**Published:** 2008-12-17

**Authors:** Kevin Kontos, Patrice Godard, Bruno André, Jacques van Helden, Gianluca Bontempi

**Affiliations:** 1 Machine Learning Group, Département d'Informatique, Faculté des Sciences, Université Libre de Bruxelles (ULB), Boulevard du Triomphe CP 212, 1050 Brussels, Belgium; 2 Physiologie Moléculaire de la Cellule, IBMM, Faculté des Sciences, ULB, Rue des Pr. Jeener et Brachet 12, 6041 Gosselies, Belgium; 3 Unité de Recherche en Biologie Cellulaire, Département de Biologie, Faculté des Sciences, Facultés Universitaires Notre-Dame de la Paix Namur (FUNDP), Rue de Bruxelles 61, 5000 Namur, Belgium; 4 Laboratoire de Bioinformatique des Génomes et des Réseaux, Faculté des Sciences, ULB, Boulevard du Triomphe CP 263, 1050 Brussels, Belgium

## Abstract

**Background:**

Nitrogen is an essential nutrient for all life forms. Like most unicellular organisms, the yeast *Saccharomyces cerevisiae *transports and catabolizes good nitrogen sources in preference to poor ones. Nitrogen catabolite repression (NCR) refers to this selection mechanism. All known nitrogen catabolite pathways are regulated by four regulators. The ultimate goal is to infer the complete nitrogen catabolite pathways. Bioinformatics approaches offer the possibility to identify putative NCR genes and to discard uninteresting genes.

**Results:**

We present a machine learning approach where the identification of putative NCR genes in the yeast *Saccharomyces cerevisiae *is formulated as a supervised two-class classification problem. Classifiers predict whether genes are NCR-sensitive or not from a large number of variables related to the GATA motif in the upstream non-coding sequences of the genes. The positive and negative training sets are composed of annotated NCR genes and manually-selected genes known to be insensitive to NCR, respectively. Different classifiers and variable selection methods are compared. We show that all classifiers make significant and biologically valid predictions by comparing these predictions to annotated and putative NCR genes, and by performing several negative controls. In particular, the inferred NCR genes significantly overlap with putative NCR genes identified in three genome-wide experimental and bioinformatics studies.

**Conclusion:**

These results suggest that our approach can successfully identify potential NCR genes. Hence, the dimensionality of the problem of identifying all genes involved in NCR is drastically reduced.

## Background

Nitrogen is an essential nutrient for all life forms. The emergence of cells able to transport, catabolize and synthesize a wide variety of nitrogenous compounds has thus been favored by evolutionary selective pressure [1]. As a consequence, the yeast *Saccharomyces cerevisiae *can use almost 30 distinct nitrogen-containing compounds [1].

Like most unicellular organisms, yeast transports and catabolizes good nitrogen sources in preference to poor ones. Nitrogen catabolite repression (NCR) refers to this selection mechanism [1,2]. More specifically, NCR inhibits the transcriptional activation systems of genes needed to degrade poor nitrogen sources [2]. All known nitrogen catabolite pathways are regulated by four regulators (Gln3, Gat1, Dal80, and Deh1) [3]. The ultimate goal is to infer the complete nitrogen catabolite pathways.

In this context, bioinformatics approaches offer the possibility to identify a relatively small number of putative NCR genes [1,2,4]. Hence, biologists need only to test a small number of "promising" candidates, instead of testing all genes, saving time and resources.

In this paper, we extend a machine learning approach [5] which has been successfully used for inferring putative NCR genes [1]. This method formulates the identification of putative NCR genes as a supervised two-class classification problem.

Compared to [1], we consider different variables. Instead of simply considering the number of occurrences of motifs that are over-represented in the upstream non-coding sequences of NCR genes, we consider a wide range of properties related to the GATA motif. This motif is important because it is recognized by the GATA family transcription factors (see [1] and references therein), which are the transcriptional regulators of NCR in *Saccharomyces cerevisiae*.

As in [1], the positive training set is composed of annotated NCR genes. Concerning the negative training set, we follow a different approach. Instead of randomly selecting genes in the yeast genome, we use a set composed of manually-selected genes known to be insensitive to NCR. Hence, our approach is less computationally expensive.

We compare three state-of-the-art classifiers, namely naive Bayes, *k*-nearest-neighbors, and support vector machine.

Given the high dimensionality of the data, we use a wrapper variable selection technique (as in [1]), and a filter approach, to improve the classifiers' performance and enhance interpretability.

The remainder of the paper is organized as follows. In the next section we first detail our approach by describing the training sets, defining the variables and presenting the classifiers and variable selection methods. Subsequently, we present the correction method we apply to the posterior probabilities returned by the classifiers. This is followed by the assessment of the classifiers' performance, the validation of the inferred putative NCR genes, and the analysis of the best ranked variables.

## Methods

### Two-class classifier

The classifier takes as input a data matrix containing *n *rows (one per gene) and *p *columns (one per variable). The *n *genes constitute the samples. The *p *variables reflect properties of the occurrences of the GATA motif in the upstream non-coding sequences of the yeast genes (see Section *Definition of variables *below). Hence, each variable is a *n*-dimensional vector. The classifier is trained on a number *n*_*t *_≪ *n *of positive and negative training samples, i.e. genes that are known to be NCR-sensitive and insensitive, respectively. The trained classifier is then used to make predictions for genes not used in the training phase.

### Training sets

As a positive training set, denoted by ANCR, we use 37 of the 41 genes previously annotated as NCR-responding [1]. Four genes are discarded because none of them were identified as NCR-responding in any of the three genome-wide experimental and bioinformatics studies described in [1,2,4]. The negative training set, denoted by NNCR, is composed of 89 manually-selected genes, known to be insensitive to NCR, most of which being involved in house-keeping cellular functions unrelated to nitrogen metabolism.

### Definition of variables

The promoter regions of NCR target genes typically contain several 5'-GATA-3' core sequences, which we will refer to as GATA boxes, recognized by the GATA family transcription factors (see [1] and references therein). Hence, the variables we define focus on the GATA boxes in the upstream non-coding sequences of the yeast genes.

Since the variables rely on the availability of the upstream non-coding sequences, we retrieved them for all yeast genes over 800 base pairs (bp) upstream from the start codon using the collection of software tools provided by the web resource Regulatory Sequence Analysis Tools (RSAT), available from [6]. When the upstream open reading frame (ORF) is closer than 800 bp, a shorter sequence is retrieved to discard coding sequences.

We now give a brief description of the 585 variables (see also Table 1 for a summary).

**Table 1:** Abbreviations and short descriptions of variables.

Abbreviation	Description
NUM	Number of GATA boxes
1-GAP, 2-GAP, 3-GAP, B-GAP	First, second and third smallest, and biggest GATA gaps
M-GAP, MI-GAP, SD-GAP	Mean, median and standard deviation (sd) of all GATA gaps
*i*-MINDIST (*i *= 2,..., 5)	Minimum number of bp spanning over *i *GATA boxes
UP-*i*-MER (*i *= 1, 2, 3)	N{1, i}GATA
DOWN-*i*-MER (*i *= 1, 2, 3)	GATAN{1, i}
GAP-*i*-MER (*i *= 1, 2)	N{1, i}GATAN{1, i}
F-POS, L-POS	Positions of the first and of the last GATA boxes, resp.
M-POS, MI-POS, SD-POS	Mean, median and sd of the positions of all GATA boxes

#### Number of GATA boxes

**Figure 1:**
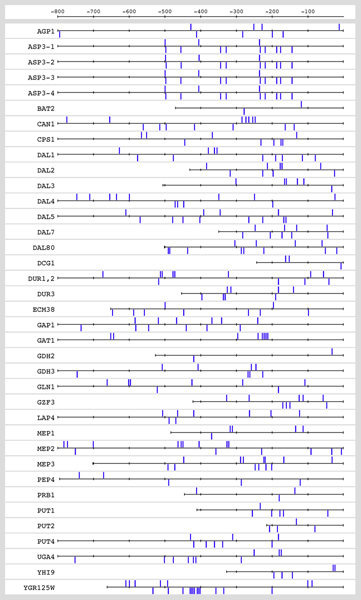
**GATA boxes in the upstream non-coding sequences of ANCR genes**. Graphical map of the GATA boxes in the upstream non-coding sequences of ANCR genes generated with RSAT [6].

**Figure 2:**
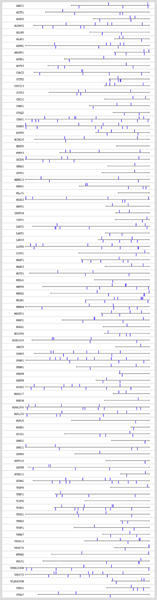
**GATA boxes in the upstream non-coding sequences of NNCR genes**. Graphical map of the GATA boxes in the upstream non-coding sequences of NNCR genes generated with RSAT [6].

As illustrated in Figures 1 and 2, the annotated NCR genes (ANCR) are characterized by a relatively large number of GATA boxes compared to genes know to be insensitive to NCR (NNCR). We therefore define a variable NUM which counts the number of GATA boxes in the upstream non-coding sequences.

#### GATA gap

Further, we note that GATA boxes often come in pairs separated by only few bp. We therefore define 11 variables related to the number of bp separating two consecutive GATA boxes in the upstream non-coding sequences, which we refer to as a GATA gap. The variables 1-GAP, 2-GAP, 3-GAP and B-GAP measure the first, second and third smallest, and biggest GATA gaps, respectively. The variables M-GAP, MI-GAP and SD-GAP measure the mean, median and standard deviation of all GATA gaps, respectively. Finally, the variables *i*-MINDIST, *i *= 2,..., 5, measure the minimum number of bp spanning over *i *GATA boxes.

#### k-mers

When searching for over-represented motifs in the upstream non-coding sequences of ANCR genes, it appears that variants of GATA boxes are relatively frequent, as for example the following motifs: GATAAG and GATAAH. Hence, we define the variables UP-*i*-MER (*i *= 1, 2, 3), DOWN-*i*-MER (*i *= 1, 2, 3) and GAP-*i*-MER (*i *= 1, 2) that count the following *k*-mers, respectively: N{1, i}GATA, GATAN{1, i} and N{1, i}GATAN{1, i}, where N{1, i} is a motif of length comprised between 1 and *i*, and where N represents any nucleotide (A, C, G or T). There are respectively 84 (= 4 + 4^2 ^+ 4^3^), 84 and 400 (= 4^2 ^+ 2 × 4^3 ^+ 4^4^) variables N{1, i}GATA, GATAN{1, i} and N{1, i}GATAN{1, i}.

#### Positions of GATA boxes

Finally, we define 5 variables relative to the positions of the GATA boxes in the upstream non-coding sequences. The position of a GATA box is defined as the number of bp separating its first bp from the start codon of the gene. The variables F-POS and L-POS measure the positions of the first (i.e., the closest to the start codon) and of the last (i.e., the farthest from the start codon) GATA boxes, respectively. The variables M-POS, MI-POS and SD-POS measure the mean, median and standard deviation of the positions of all GATA boxes, respectively.

### Classifiers

We compare three classifiers, namely naive Bayes (NB) [7], *k*-nearest-neighbors (KNN) [7], where leave-one-out error is used to choose the number of neighbors, and linear kernel support vector machine (SVM) [8].

### Variable selection

Because of the high-dimensionality of the classification task, i.e., the number of variables is greater than the number of samples, we compare two variable selection methods to improve prediction performance and enhance interpretability.

First, we use a filter method [9] based on the Gram-Schmidt orthogonalization procedure [8] where the number of selected variables is determined according to leave-one-out cross-validation [10]. The ranking of variables through orthogonalization has many interesting features: it is computationally fast, it takes into account the collinearity between variables (i.e., if two variables are almost collinear in observation space, the fact that one of them is selected will tend to drive the other to a much lower rank in the list) and it allows an incremental construction of the model, so that training can be terminated without using all variables [11]. Although this method assumes linearity and is based on the minimization of a squared error loss (which is not always the most appropriate for classification), it gives relatively good results for classification tasks [11].

Second, we use a wrapper method [12] consisting of a forward stepwise procedure where the prediction performance is assessed by means of stratified 10-fold cross-validation [8]. The performance measure used is the balanced error rate (BER, see Section *Results and Discussion *below for its definition) and the threshold on the corrected posterior probability (see next section) is 0.5 By using the prediction performance of a given learning machine to assess the relative usefulness of subsets of variables, wrappers offer a simple and powerful way to address the problem of variable selection [9,12]. A greedy search strategy, such as forward selection, is both computationally advantageous and robust against overfitting [9].

### Posterior probability correction

The classifiers provide estimates of the posterior probabilities that rely on the prior probabilities of the training set. Unfortunately, these prior probabilities do not reflect the expected prior probabilities of the target classes. Therefore, we adjust the posterior probabilities returned by the classifiers with respect to new prior probabilities by Bayes' theorem [13]. These new priori probabilities are chosen according to prior biological knowledge: more or less 200 genes are expected to be targets of NCR [1]. Hence, we set the prior probability of a gene to be target of NCR to $\frac{200}{n}$, where *n *= 5869 is the total number of yeast genes considered.

## Results and discussion

### Validation

We assess the quality of the variable selection methods and classifiers through leave-one-out cross-validation. We use two performance measures:

• The balanced error rate (BER), defined as the average of the errors on each class. The threshold on the corrected posterior probability is 0.5. Results are shown in the "BER" column of Table 2. The best combinations of variable selection method and classifier, i.e., those having a BER not significantly higher than the lowest BER according to McNemar's test [14] with *P*-value < .05, are marked with an asterisk (*).

**Table 2:** Performance assessment. VS and CLASS stand for variable selection method and classifier, respectively.

					Negative control		
			
VS	CLASS	BER	AUC	AUCext	BER	AUC	AUCext
Filter	NB	0.31	0.93	0.95	0.49 ± 0.022	0.50 ± 0.072	0.63 ± 0.023
	KNN	0.18	0.90	0.91	0.51 ± 0.021	0.51 ± 0.077	0.34 ± 0.088
	SVM	0.13*	0.93	0.98	0.48 ± 0.060	0.50 ± 0.097	0.67 ± 0.026
	NB	0.24	0.95	0.91	0.49 ± 0.054	0.50 ± 0.130	0.48 ± 0.016

Wrapper	KNN	0.20	0.97	0.66	0.48 ± 0.045	0.52 ± 0.100	0.41 ± 0.073
	SVM	0.13*	0.95	0.88	0.47 ± 0.066	0.58 ± 0.130	0.58 ± 0.042

• The area under the receiver operator characteristic (ROC) curve (AUC). The use of ROC curves is recommended when evaluating binary decision problems in order to avoid effects related to the chosen threshold on the posterior probabilities [15]. Results are shown in the "AUC" column of Table 2.

#### Extending the "gold standard"

We used the ANCR and NNCR sets as a "gold standard" in the validation step (through leave-one-out cross-validation since these sets are also used to train the classifiers). We now extend, in the validation step (but not in the training phase), the set of "true" NCR genes with the putative NCR genes identified in three genome-wide experimental and bioinformatics studies [1,2,4]. Hence, the "true" NCR genes in the validation step are composed of the ANCR genes and the genes provided by the three experimental studies.

The quality of the predictions are evaluated according to the AUC. Results are shown in the "AUCext" column of Table 2.

#### Negative control

Given the scarcity of the data and the risk of the variable selection procedure to overfit the selected variables to the training set, we perform a negative control to determine whether the results are significant or not. We empirically estimate the random rate of correct classification by running the same procedure but with randomized data sets obtained by randomly sampling the labels of the training set. Results are shown in the "negative control" columns of Table 2. The values reported are the mean and standard deviation over 10 repetitions.

### Gene set comparisons

For each combination of variable selection method and classifier, we compare the set of predicted NCR genes, obtained with a threshold of 0.5 on the corrected posterior probability, with each of the three sets identified in the three aforementioned studies [1,2,4], respectively. More specifically, we compute for each combination of variable selection method and classifier, and for each set, the *F*-measure defined as the harmonic mean of the precision and recall quantities:

$$F(prec,rec)=\{\begin{array}{ll} \frac{2\cdot prec\cdot rec}{prec+rec} & \text{if }prec+rec>0;\\ 0 & \text{otherwise}. \end{array}$$

The precision quantity measures the fraction of true positives among those inferred as positive:

$$prec=\{\begin{array}{ll} \frac{TP}{TP+FP} & \text{if }TP+FP>0;\\ 0 & \text{otherwise}; \end{array}$$

and the recall quantity measures the fraction of true positives among all "true" NCR genes:

$$rec=\{\begin{array}{ll} \frac{TP}{TP+FN} & \text{if }TP+FN>0;\\ 0 & \text{otherwise}\text{.} \end{array}$$

Results are shown in Table 3.

**Table 3:** Gene set comparisons. VS and CLASS stand for variable selection method and classifier, respectively.

		*F*-measure (*P*-value)		
		
VS	CLASS	Bar-Joseph et al., 2003 [4]	Godard et al., 2007 [1]	Scherens et al., 2006 [2]
Filter	NB	0.05 2.9 × 10^-16^	0.09 (3.5 × 10^-7^)	0.06 (2.4 × 10^-13^)
	KNN	0.06 (9.4 × 10^-9^)	0.09 (4.8 × 10^-5^)	0.07 (1.1 × 10^-7^)
	SVM	0.11 (1.5 × 10^-13^)	0.15 (9.0 × 10^-10^)	0.14 (8.2 × 10^-14^)

Wrapper	NB	0.07 (9.1 × 10^-11^)	0.11 (7.7 × 10^-18^)	0.08 (4.3 × 10^-16^)
	KNN	0.12 (7.7 × 10^-14^)	0.20 (7.0 × 10^-28^)	0.16 (5.2 × 10^-26^)
	SVM	0.13 (8.9 × 10^-11^)	0.16 (7.2 × 10^-14^)	0.13 (2.6 × 10^-11^)

#### Negative control

To assess the significance of the overlap between two sets, and to account for the artificial increase in the overlap that occurs with increasing number of predicted NCR genes (i.e., with decreasing threshold on the corrected posterior probability), we also compute overlapping *P*-values on the basis of the cumulative distribution function of the hypergeometric distribution [1]. Results are shown in Table 3.

### Variable selection

The improvement of prediction performance with variable selection is confirmed by the number of variables returned by the wrapper approach. Indeed, for all classifiers, the number of selected variables is small (in the order of tens) compared to the total number of variables (585).

The top selected variables are *k*-mers (see UP-*i*-MER, DOWN-*i*-MER and GAP-*i*-MER in Table 1). More specifically, the following motifs were (almost) always selected: GATAAG, TAGATAA, GATAGG and GTAGATA. The GATAAG motif is known to be potentially relevant for the NCR regulation [3,16]. Analysis of the other motifs is ongoing.

## Conclusion

We proposed a machine learning approach where the identification of putative NCR genes in the yeast *Saccharomyces cerevisiae *is formulated as a supervised two-class classification problem.

Based on almost 600 variables, we showed that all classifiers made significant and biologically valid predictions by comparing the predictions to annotated and putative NCR genes, and by performing several negative controls. In particular, the inferred NCR genes significantly overlap with putative NCR genes identified in three genome-wide experimental and bioinformatics studies [1,2,4].

These results suggest that our approach can successfully identify potential NCR genes. Hence, the dimensionality of the problem of identifying all genes involved in NCR is reduced, saving time and resources.

Although all classifiers produced significant results, McNemar's test suggests that the linear kernel support vector machine performs best (independently of the variable selection method).

In order to thoroughly evaluate the proposed approach, the putative NCR genes identified will be tested in vitro for NCR-sensitivity. We also plan to extend this approach to other yeast species to study the evolution of NCR.

## Competing interests

The authors declare that they have no competing interests.

## Authors' contributions

K.K. and G.B. developed the methodology. K.K. carried out all experiences and analyses, and wrote the manuscript. P.G., B.A. and J.v.H. defined the biological problem, provided the data and validated the results. All authors contributed to, read and approved the final version of the manuscript.
